# Prevalence and predictors of NTM in presumed/confirmed drug-resistant TB

**DOI:** 10.5588/ijtldopen.24.0242

**Published:** 2024-07-01

**Authors:** E.T. Abbew, R. Laryea, F. Sorvor, Y.A. Poku, N. Lorent, D. Obiri-Yeboah, L. Lynen, L. Rigouts

**Affiliations:** ^1^ Department of Clinical Sciences, Institute of Tropical Medicine, Antwerp, Belgium;; ^2^ Department of Internal Medicine, Cape Coast Teaching Hospital, Cape Coast, Ghana;; ^3^ Department of Biomedical Sciences, University of Antwerp, Belgium;; ^4^ Eastern Regional Hospital, Koforidua, Ghana;; ^5^ National Tuberculosis Control Programme, Accra, Ghana;; ^6^ Department of Respiratory Diseases, University Hospital Leuven, Leuven, Belgium;; ^7^ Department of Chronic Diseases, Metabolism and Aging, BREATHE laboratory, Katholieke Universiteit Leuven, Leuven, Belgium;; ^8^ Department of Microbiology and Immunology, School of Medical Sciences, University of Cape Coast, Ghana;; ^9^ Department of Biomedical Sciences, Institute of Tropical Medicine, Antwerp, Belgium

**Keywords:** non-tuberculous mycobacteria, non-tuberculous mycobacterial pulmonary disease, drug-resistant tuberculosis

## Abstract

**INTRODUCTION:**

Non-tuberculous mycobacteria (NTM) are increasingly isolated in individuals with presumed/confirmed pulmonary TB. We aimed to estimate the prevalence and species distribution of NTM among presumed/confirmed drug-resistant TB (DR-TB) individuals and determine NTM isolation predictors.

**METHODS:**

Sputum samples collected for DR-TB diagnosis and follow-up from 2012 to 2021 in Ghana were retrospectively analysed. Samples were subjected to sputum smear microscopy (SSM) and mycobacterial culture. The MPT64 assay was performed on positive cultures to distinguish between *Mycobacterium tuberculosis* complex MTBc and NTM. NTM isolates were re-cultured for species identification using GenoType® Mycobacterium CM/AS line-probe assay, polymerase chain reaction, and Sanger sequencing targeting 16S rRNA and *rpo*B genes. MTBc isolates identified by GenoType underwent spoligotyping. A logistic regression model was used to identify the predictors of NTM isolation.

**RESULTS:**

Of the 2,492 samples, 839 (33.7%) tested culture-positive for mycobacteria, with 257 (30.6%) presumed to be NTM. Of these, 53 (23.6%) were identified at the species level, with a predominance of *M. intracellulare* (66.0%). MPT64 testing missed 18 (3%) MTBc isolates. Logistic regression showed increased odds of NTM isolation in follow-up samples (aOR 2.41, 95% CI 1.46–3.99). NTM species were isolated from 46 patients, with four classified as NTM pulmonary disease

**CONCLUSION:**

Enhancing our understanding of local NTM epidemiology and improving local diagnostic capabilities can optimise patient management strategies and outcomes.

## RÉSUMÉ

**INTRODUCTION:**

Les mycobactéries non tuberculeuses (NTM) sont de plus en plus souvent isolées chez les personnes atteintes de TB pulmonaire présumée/confirmée (DR-TB). Notre étude visait à évaluer la fréquence et la répartition des différentes espèces de NTM chez les personnes atteintes de TB pharmacorésistante, ainsi qu'à identifier les facteurs prédictifs de l'isolement de ces NTM.

**MÉTHODES:**

Les échantillons d'expectorations collectés entre 2012 et 2021 au Ghana pour le diagnostic et le suivi de la DR-TB ont été analysés rétrospectivement. Les échantillons ont subi une microscopie du frottis d'expectoration (SSM) et une culture mycobactérienne. Le test MPT64 a été réalisé sur les cultures positives pour différencier le complexe *Mycobacterium tuberculosis* (MTBc) et les NTM. Les isolats de NTM ont été soumis à une nouvelle culture pour identification des espèces à l'aide du test par sondes en ligne GenoType^®^ Mycobacterium CM/AS, de l'amplification en chaîne par polymérase et du séquençage de Sanger ciblant les gènes 16S rRNA et *rpo*B. Les isolats de MTBc identifiés par GenoType ont été soumis à un spoligotypage. Un modèle de régression logistique a été utilisé pour identifier les facteurs prédictifs de l'isolement des NTM.

**RÉSULTATS:**

Parmi les 2 492 échantillons analysés, 839 (33,7%) ont été testés positifs à la culture de mycobactéries, dont 257 (30,6%) étaient présumés être des NTM. Parmi ces échantillons, 53 (23,6%) ont été identifiés au niveau de l'espèce, avec une prédominance de *M. intracellulare* (66,0%). Le test MPT64 a échoué à détecter 18 (3%) isolats de MTBc. L'analyse de régression logistique a révélé une probabilité accrue d'isolement de MNT dans les échantillons de suivi (aOR 2,41 ; IC à 95% 1,46–3,99). Des espèces de NTM ont été isolées chez 46 patients, dont quatre ont été classés dans la catégorie des maladies pulmonaires à NTM.

**CONCLUSION:**

Une connaissance approfondie de l'épidémiologie locale des NTM et le renforcement des compétences de diagnostic au niveau local peuvent améliorer les stratégies de prise en charge des patients et les résultats obtenus.

In recent years, non-tuberculous mycobacteria (NTM) have increasingly been isolated from pulmonary samples of people undergoing evaluation for pulmonary TB globally.^1,2^ This suggests that a proportion of individuals initially presumed to have TB harboured NTM infections in their lungs.^3^ The isolation of NTM in such samples highlights the importance of distinguishing between NTM and *Mycobacterium tuberculosis* complex (MTBc), as their disease requires different management strategies and treatments.^4,5^ Accurate identification of NTM in pulmonary specimens is a crucial first step for appropriate patient care, preventing unnecessary TB treatment, and ensuring proper management of NTM-related lung disease.^6^

The prevalence and diversity of NTM vary globally. In North America, *M. avium* complex (MAC) and *M. abscessus* complex are predominant, especially in structural lung disease.^7^ Europe sees similar diversity with *M. avium* complex, *M. xenopi*, and *M. kansasii* causing infections.^8^ In Asia*, M. fortuitum* and *M. intracellulare* are common.^9,10^ Geographical differences highlight the interplay of environmental factors and host susceptibility.

NTM are environmental mycobacteria, and determining their clinical significance can be challenging. NTM growth in the laboratory can indicate potential environmental contamination or transient passage in the lungs. This can be effectively prevented by employing laboratory standard operating procedures.^11^ Sputum samples for mycobacterial culture should be collected using early morning samples and transported to the laboratory for culture and subsequent speciation. All infection prevention procedures must be adhered to limit environmental contamination.^12^

An isolated NTM is deemed clinically significant when the same species is repeatedly isolated from a pulmonary sample and when there are clinical and radiological signs and symptoms compatible with pulmonary disease and no other explanation can be found.^13^ The American Thoracic Society/European Respiratory Society/European Society of Clinical Microbiology and Infectious Diseases/Infectious Diseases Society of America has proposed an international guideline that combines clinical symptoms, mycobacterial isolation, and imaging findings to diagnose NTM pulmonary disease (NTM-PD).^5^ Identification of NTM species is a crucial step in determining their clinical significance. In the sub-Saharan African (SSA) region, NTM-PD has been observed in individuals previously undergoing treatment for recurrent TB.^14^ Additionally, a history of prior TB treatment has been recognised as a significant risk factor for NTM-PD.^15,16^ Notably, within Africa, a considerable proportion of individuals under investigation for drug-resistant TB (DR-TB) have a documented history of previous TB treatment.^17^ Hence, the risk factors for NTM-PD and DR-TB are similar in SSA.

Therefore, we aimed to estimate the prevalence of NTM among the sputum samples of individuals with presumed or confirmed DR-TB in Ghana between 2012 and 2021, assess the trend of NTM isolation, describe the NTM species, and determine the predictors of NTM isolation.

## METHODS

### Study population and variables

The study included patients with presumed or confirmed DR-TB who provided sputum samples during the programmatic work-up for diagnosis/follow-up of DR-TB. A patient can have multiple samples at various points in the treatment cycle. Each sample is counted as one unit. Routine data of programmatic sample collection from 2012 to 2021 were used in this analysis. Samples were gathered from 12 of the 16 regions in Ghana. To facilitate analysis, these regions were categorised into three groups: the “Southern Belt,” which includes the Western, Greater Accra, Volta, Central, and Eastern regions; the “Middle Belt,” consisting of Ashanti, Bono, Bono East, Oti, and Western North regions; and the “Northern Belt,” comprising the North East and Northern regions.

Variables collected during the routine laboratory data collection process include age, sex, region, and the reason for the investigation (diagnosis and follow-up).

### Sputum culture and storage of isolates

Sputum samples were collected using the concentration method and analysed through sputum smear microscopy (SSM). Next, a culture using the BACTEC Mycobacteria Growth Indicator Tube (MGIT) 960 was used to identify mycobacterial growth. A blood agar plate was inoculated with tubes flagging positive to track contamination. Differentiation of MTBc from NTM was performed using the MPT64 assay (SD Bioline, Seoul, South Korea), a rapid diagnostic test targeting the MPT64 protein specific to MTBc. The presence of acid-fast bacilli (AFB) in the positive MGIT tubes by microscopy was not confirmed. Phenotypic drug-susceptibility testing (DST) was performed on MPT64-confirmed MTBc isolates. Mycobacterial isolates that remained negative in the MPT64 assay were considered “presumed NTM” and stored at -20^0^C for up to 10 years. All isolates were pseudonymised.

### Species identification using GenoType^®^ Mycobacterium CM/AS

In 2022, the stored presumed NTM isolates were re-cultured in the MGIT960 system, and those that were successfully re-grown were species-typed using the line probe assay (LPA) GenoType® Mycobacterium CM/AS (Hain Lifesciences, Germany) as per manufacturer's instructions at the Eastern Regional Hospital in Ghana.

### PCR and Sanger sequencing

The presumed NTM isolates identified as “*Mycobacterium* species” and those unidentified by GenoType CM/AS assays were further analysed using PCR and Sanger sequencing targeting the 16S rRNA and *rpoB* genes.^18,19^

### Spoligotyping

Spoligotyping was performed using in-house prepared membranes for the isolates identified as MTBc by the GenoType CM assay.^20^ The spoligotyping results were compared with the publicly available profiles in the mycobacterial interspersed repetitive units (MIRU) variable number of tandem repeats (VNTR)- plus database (www.miru-vntrplus.org/MIRU/index.faces) to identify the lineages using a similarity search.

### Statistical analysis

All data were captured in an Excel spreadsheet. Descriptive statistics include the mean and standard deviation for numerical variables and percentages for categorical variables. A logistic regression model was fitted using the predictors of NTM isolation and the covariates of age, sex, smear microscopy, and reason for examination (Table 1). All analyses were performed using STATA version 18 (Stata Corp, Texas, USA).

**Table 1.** Sociodemographic characteristics of population cohort and NTM speciation.

Characteristics		NTM only
	Total population	(*n* = 53)
	*n* (%)	*n* (%)
Age, years, mean ± SD (*n* = 2,375)*	45 ± 14.9	45 ± 12.9
<15	59 (2.5)	1 (1.9)
15–29	310 (13.1)	4 (7.5)
30–44	777 (32.7)	19 (35.8)
45–59	911 (38.4)	24 (45.3)
≥60	318 (13.4)	5 (9.4)
Sex (*n* = 2,486)*		
Male	1,686 (67.8)	37 (69.8)
Female	800 (32.2)	16 (30.2)
Region (*n* = 2,490)*		
Southern Belt^†^	2,117 (85.0)	42 (79.2)
Middle Belt	370 (14.9)	11 (20.8)
Northern Belt	3 (0.1)	0 (0.0)
Reason for investigations (*n* = 2,438)*		
Diagnosis	1,004 (41.2)	15 (28.3)
Follow-up	1,434 (58.8)	38 (71.7)
NTM species identification (*n* = 53)		
*M. intracellulare*		35 (66.0)
*M. fortuitum*		11 (20.7)
*M. abscessus* complex		2 (3.8)
*M. malmoense*		2 (3.8)
*M. parascrofulaceum*		1 (1.9)
*M. shigaense*		1 (1.9)
*M. avium*		1 (1.9)

* Total population.

† The predominance of the southern belt reflects the patient distribution of DR-TB in Ghana.

NTM = non-tuberculous mycobacteria; SD = standard deviation; DR-TB = drug-resistant TB.

### Ethics

The study received ethical approval from Cape Coast Teaching Hospital (CCTHERC/EC/2022/156) and the Institute of Tropical Medicine (I632/22), and permission was obtained from the National Tuberculosis Control Programme. An informed consent waiver was obtained because no prospective patient-level data were captured. All persons undergoing management (investigation and treatment) in Ghana provided informed consent.

## RESULTS

### Sample processing and species identification

**Figure 1.**
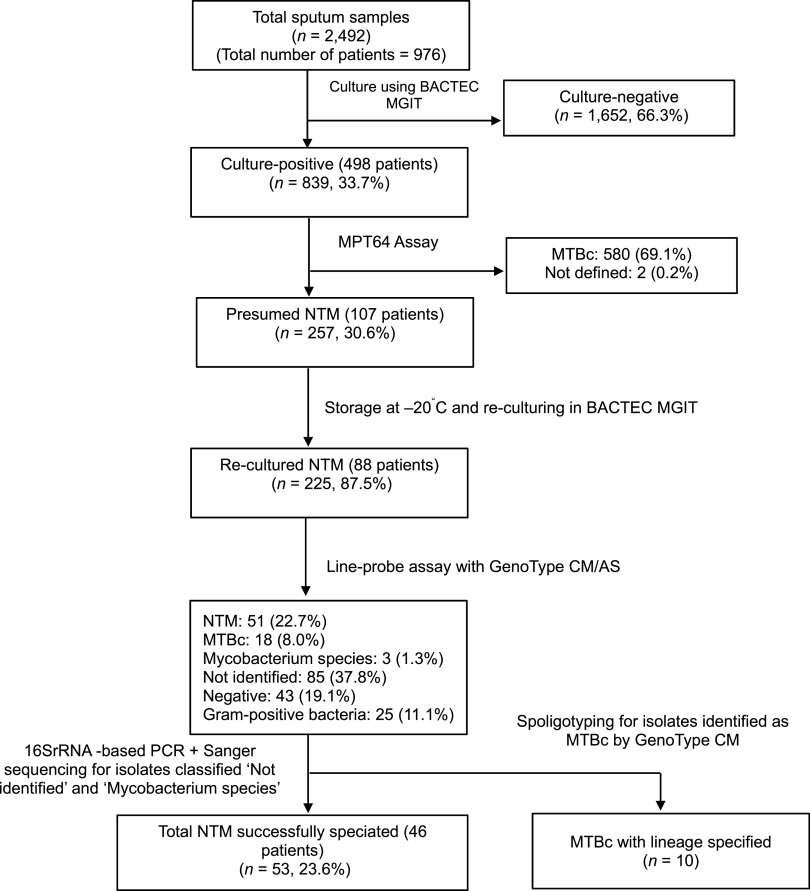
Flowchart of analysis of sputum specimens and presumed NTM culture isolates. NTM = non-tuberculous mycobacteria; MGIT = Mycobacteria Growth Indicator Tube; MTBc = *M. tuberculosis* complex; PCR = polymerase chain reaction.

Figure 1 depicts a flowchart of sample analysis that illustrates the processing of 2,492 sputum samples from 976 patients collected between 2012 and 2021. Of these, 839 samples (33.7%) from 498 patients tested culture positive. Among the culture-positive samples, 257 (30.6%) from 107 patients were presumed to be NTM based on negative MPT64 antigen results and were stored at 20°C. Of these, 225 samples from 88 patients were positive for MGIT when regrown from the collection.

After GenoType® Mycobacterium CM/AS, 51 (22.7%) of the presumed NTM isolates were successfully identified to the species level (Figure 1), while three were confirmed as belonging to the genus *Mycobacterium* but not one of the species comprised in the assay, 25 (11.1%) were identified as Gram-positive bacteria, 85 (37.8%) could not be identified, and 43/225 (19.1%) were negative. GenoType® Mycobacterium CM/AS identified MTBc in 18 (18.0%) of 225 regrown isolates, which initially remained negative by the MPT64 antigen test and were erroneously interpreted as “presumed NTM”.

After further PCR and sequencing analysis, 53 (23.6%) of the 225 presumed NTM isolates were identified at the species level. Among the identified NTM species, *M. intracellulare* was predominant, accounting for 66.0% of the identified species, followed by *M. fortuitum* (20.7%) (Table 1). The remaining species included *M. abscessus* complex, *M. malmoense*, *M. parascrofulaceum*, *M. shigaense*, and *M. avium* complex.

### Trend and distribution of NTM isolates

**Figure 2.**
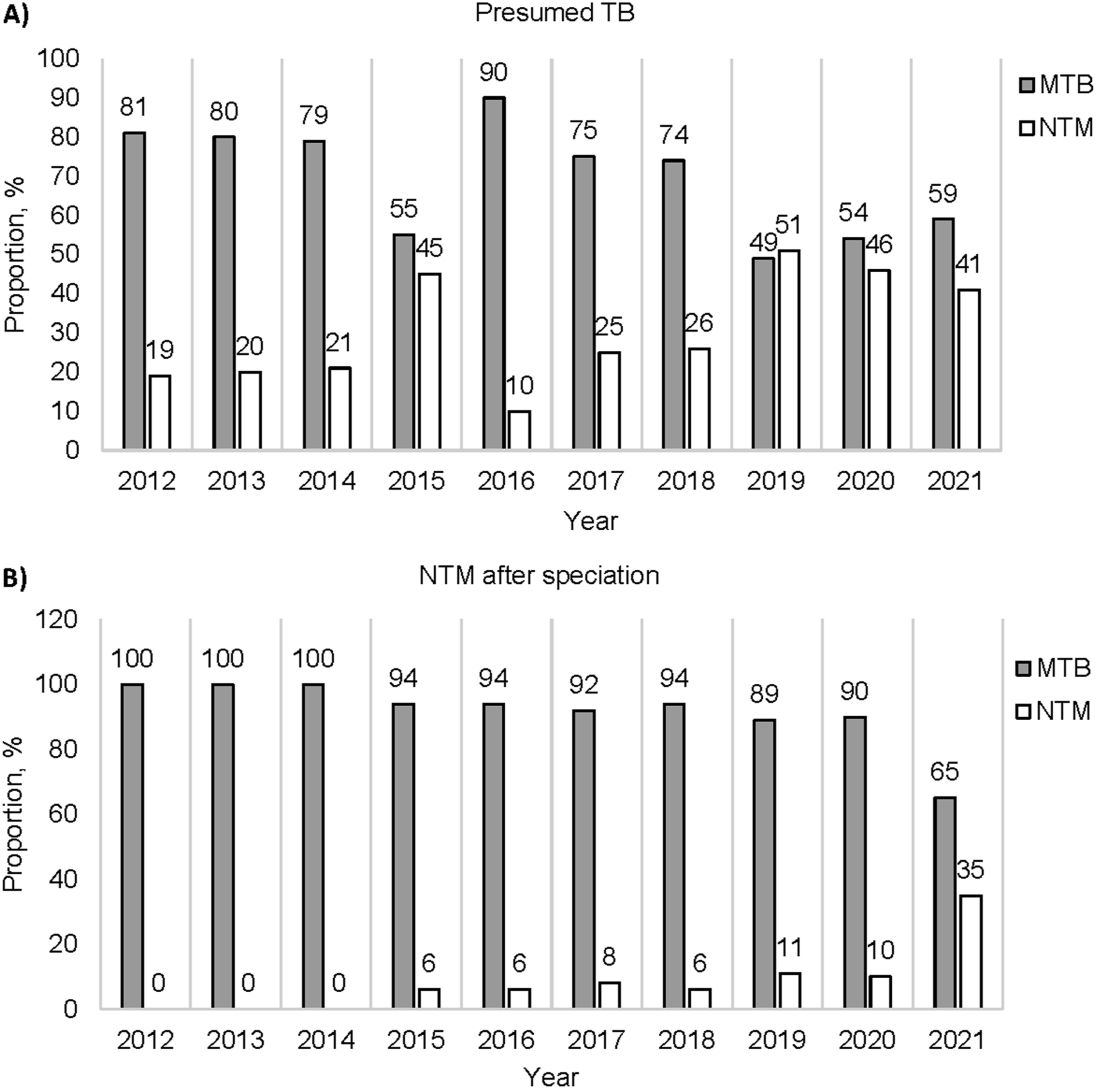
Proportion of MTB complex and NTM based on **A)** initial MGIT culture and MPT64 antigen testing, or **B)** final NTM identification by molecular methods. MTB = *M. tuberculosis*; NTM = non-tuberculous mycobacteria; MGIT = Mycobacteria Growth Indicator Tube.

Initially, 10% of the total sample 2492 was identified as “presumed NTM” based on culture and MPT64 testing. However, upon further molecular speciation, only 2% of the samples were identified as harbouring NTM species (Figures 2A and 2B). Interestingly, the final identification results showed a trend of higher NTM yield in more recent years, with no NTMs for 2012-2014, 6.0-11.0% from 2015 to 2020, and 35.0% in 2021 (Figure 2B).

### MTBc missed by initial MPT64 testing

Eighteen (3.3%) of the final identified 598 MTBc were missed by initial MPT64 testing. Of these, 14 isolates were available for spoligotyping, of which 10 were successfully typed, whereas the remaining had insufficient DNA extract for reliable lineage typing (Supplementary Table S1). Among the successfully typed isolates, five were identified as *M. tuberculosis* Cameroon, belonging to lineage 4 (L4), three as *M. tuberculosis* Ghana (L4), and two as *M. africanum* West africanum type 1 (L5).

### Sociodemographic characteristics and predictors of NTM isolation and NTM-PD

Of all sputum samples collected, 1,686 (67.8%) were from males (Table 1), with the average age of patients providing samples being 45 years (SD 14.9). Less than half of the samples (n=1,004; 41.2%) were collected for the diagnostic purposes of DR-TB. Age and sex distributions were similar for the NTM cohort, with male predominance (*n* = 37, 69.6%). Samples from the ‘Southern belt' region comprised 85.0% of the total and 79.2% of the NTM-positive samples, respectively. The predominance of the Southern belt reflects the distribution of DR-TB patients in Ghana.

The odds of having NTM isolated were significantly higher among individual samples undergoing follow-up examinations compared with those having a sample analysed for DR-TB diagnostic purposes (OR 5.81, 95% CI 3.12–10.84; aOR 2.41, 95% CI 1.46–3.99). However, when adjusted for confounders, the odds of NTM isolation for sex, age category, and smear status were not statistically significantly different (Table 2).

**Table 2.** Predictors of NTM isolation.

Predictors	MTB *n* (%)		NTM *n* (%)		OR (95% CI)		aOR (95% CI)	
Sex								
Male	436	66.9	37	5.7	Reference		Reference	
Female	163	25.0	16	2.5	1.16	0.63–2.13	0.37	0.20–0.67
Smear status								
Positive	359	55.1	10	1.5	0.16	0.08–0.32		
Negative	240	36.8	43	6.6	Reference			
Reason for examination								
Diagnosis	406	63.8	15	2.4	Reference		Reference	
Follow up	177	27.8	38	6.0	5.81	3.12–10.84	2.41	1.46–3.99
Age category, years								
Young (<30)	99	15.8	5	0.8	Reference		Reference	
Old (≥30)	476	75.8	48	7.6	1.99	0.77–5.14	0.28	0.18–0.44

NTM = non-tuberculous mycobacteria; MTB = *M. tuberculosis*; OR = odds ratio; CI = confidence interval; aOR = adjusted OR.

NTM species were isolated from 46 patients, of whom four had the same NTM species isolated at multiple time points, making them eligible for classification as NTM-PD (Table 3). All four patients were diagnosed with rifampicin-resistant TB (RR-TB) using the Xpert MTB/RIF assay (two with a high level of MTB detected, two with very low levels of detected MTB), with three having a history of first-line TB treatment.^21^ Culture results indicated that three of the four patients had consistent identification of the same NTM species in two follow-up samples. One patient exhibited NTM presence in the baseline and one follow-up sample (Table 3). Two patients were treated with a bedaquiline-containing drug regimen lasting for 9 and 11 months. The other core drugs included levofloxacin, clofazimine, high-dose isoniazid, and linezolid. The other two patients were treated with capreomycin and a levofloxacin- or moxifloxacin-containing regimen lasting 18 months. All four patients experienced a successful outcome in the treatment of their DR-TB. Only one of the four patients was HIV positive (Table 3).

**Table 3.** Summary of patient characteristics qualifying to be evaluated for NTM-PD.

Age, sex, occupation	Drug resistance (TB type and Xpert results)	HIV status	NTM species identified and month of isolation	Other culture results	Previous TB treatment	Outcome of previous treatment	DR-TB regimen	Outcome of DR-TB treatment
50, female, unemployed	RR-TB (MTB detected, very low, RR-TB detected)	Negative	*M. intracellulare* (follow-up month: 2, 8, 9)	Baseline: MTB R: resistant H, Km, Cpm, Lfx: susceptible Month 1, 3, 5, 6, 7: negative	Yes, 2RHZE/4RH	Completed	Z, E, Cpm, Mfx, Cfz, Pto, Hh (18 months)	Completed
29, male, unemployed	RR-TB (MTB detected – high, RR-TB detected	Negative	*M. intracellulare* (follow-up month: 8, 9)	Baseline: not available Month 1, 3, 11: negative Month 4, 6, 10: contaminated	Yes, 2RHZE/4RH	Lost to follow-up	E, Z, Lfx, Pto, Hh, Cfz, Bdq (11 months)	Cured
53, male, security personnel	RR-TB (MTB detected – very low, RR-TB detected)	Negative	*M. intracellulare* (follow-up month: 4, 5)	Baseline: negative Month 1, 2, 3, 6, 7, 8, 9: negative	No	Not applicable	E, Z, Cfz, Lfx, Bdq, Pto, Hh, Lzd (9 months)	Cured
61, female, trader	RR-TB (MTB detected – high, RR-TB detected)	Positive	*M. fortuitum* (diagnosis, follow-up month: 11)	Baseline: M. fortuitum Month 3, 12: MTB Month 1, 2, 4, 5, 6, 7, 8, 9, 13: negative	Yes, 2RHZE/4RH	Failure	Z, E, Cm, Lfx, Cfz, Pto, Hh (18 months)	Completed

NTM-PD = non-tuberculous mycobacteria pulmonary disease; DR-TB = drug-resistant TB; RR-TB = rifampicin-resistant TB; MTB = *Mycobacterium tuberculosis*; R = rifampicin; H = isoniazid; Km = kanamycin; Cpm = capreomycin; Lfx = levofloxacin; Z = pyrazinamide; E = ethambutol; Mfx = moxifloxacin; Cfz = clofazimine; Pto = protionamide; hH = high-dose isoniazid; Bdq = bedaquiline; Lzd = linezolid.

## DISCUSSION

Our findings shed light on identifying and classifying potentially clinically important NTM for clinical decision-making in Ghana. We identified *M. intracellulare, M. fortuitum*, *M. abscessus* complex, and *M. avium* complex, all NTM species that have also been shown to be potentially pathogenic in SSA and, in some cases, fulfil the criteria of NTM-PD.^14–16^ In our study, we found 46 presumed DR-TB patients with NTM isolates, of which four could be identified as potentially having NTM-PD, with the same NTM species isolated from two or more sputum specimens. All four persons had been diagnosed with RR-TB by GeneXpert, but three persons had NTM isolated at different months during the TB treatment follow-up period. NTM-PD is an orphan disease in SSA. Patients present with symptoms similar to those of TB, are often SSM-positive, and are at risk of being labelled as recurrent TB, thereby being treated repeatedly with antituberculosis medications. This increases the cost of the public health system and reduces the quality of life of the affected persons.^14,22^ The affected persons were successfully treated with a bedaquiline- or fluoroquinolone-based DR-TB regimen. We could not assess all international criteria to classify as NTM-PD as their imaging findings could not be retrieved. Although most pathogenic NTM species are resistant to anti-TB medicines, drugs such as bedaquiline, linezolid, and aminoglycosides have been shown to have potential activity against NTM.^23^

Overall, we could not assess the contribution of isolated NTM for all persons, as most people (800/976, 82.0%) had only a single sputum tested for culture. Nevertheless, the odds of having NTM isolated were significantly higher among individual samples undergoing follow-up examinations compared with those having a sample analysed for DR-TB diagnostic purposes (OR: 5.81, 95%CI 3.12-10.84, aOR: 2.41, 95% CI 1.46-3.99). Studies conducted in SSA have enumerated previous TB as a major risk factor for NTM isolation in patient populations.^15,16^ This finding helps to raise awareness of NTM following TB treatment and support national TB programmes through comprehensive diagnostic work-up, including NTM, to avoid unnecessary DR-TB treatment.

Furthermore, the initial 10% presumed NTM among positive MGIT cultures appeared to be an overestimation, which was corrected to 2% using molecular methods. Molecular NTM species identification remains integral to the ATS/ERS/ESCMID/IDSA criteria for diagnosing NTM-PD.^5^ Incorporating molecular NTM species identification into the routine workflow—potentially as an add-on test to the MPT64 assay for practical and financial reasons—is crucial for improving clinical decision-making and patient follow-up.^16,24^ Our study observed a reduced yield of NTM growth and speciation in older stored samples, suggesting that the viability and growth potential of NTM may decline with prolonged sample storage, with potentially also suboptimal (non-documented) storage conditions or suboptimal initial viability of the stored suspension. Thus, on-the-spot species typing with LPA from MGIT offers a promising solution for increasing the yield of accurate species identification. However, it must be noted that LPA technology for MTB DST is increasingly being phased out in favour of cartridge-based low-complexity assays.^25,26^ This may jeopardise further deployment and LPA roll-out for NTM species identification.

By employing molecular identification methods, we highlight instances where members of MTBc were initially misclassified as NTM due to the failure of the MPT64 assay to detect them. The challenge of using the MPT64 assay for MTBc lineages L5 and L6 (*M. africanum* 1 and 2), which have low or delayed MPT64 production, has been identified in West African settings.^27,28^ In our study, however, only 20% of these MPT64-negative MTBc isolates that could be typed were L5, while the remaining belonged to L4 (Cameroon and Ghana family), which are usually identifiable by the MPT64 assay.^27,28^ This shows the importance of employing an assay that identifies MTBc with greater sensitivity, as missed MTBc isolates will not undergo DST and may impact patient care.

Our study has potential limitations. First, only stored presumed NTM isolates were analysed. This could limit the study findings' representativeness, as not all DR-TB patients may have had culture done at the time of diagnosis. Second, we only analysed NTM isolates that were regrown in the laboratory. This implies that some NTM isolates, particularly those that are difficult to grow or take longer to culture, may have been missed or excluded from the analysis. This limitation could impact the comprehensiveness of the results.

## CONCLUSION

In conclusion, our study highlights important findings regarding isolating and characterising potentially clinically significant NTM species. *M. intracellulare* predominates among routine TB programme samples in Ghana, which indicates its potential clinical relevance and local prevalence in this specific geographic region.

One significant challenge in our study was the high proportion of NTM isolates that could not be identified using LPAs. This shows the importance of employing additional molecular methods of NTM identification in SSA regions in routine programmatic settings to enhance clinical decision-making. It is worth noting that our study revealed instances where members of the MTBc were initially misclassified as NTM. This emphasises the importance of employing rigorous and comprehensive diagnostic algorithms that encompass molecular identification techniques, such as LPA and molecular sequencing, to differentiate between NTM and MTBc species accurately. Misclassification can lead to delayed or inappropriate treatment, underscoring the need for improved accuracy in NTM identification.

By improving our understanding of local NTM epidemiology and enhancing diagnostic capabilities, we can optimise patient management treatment strategies and contribute to improved health outcomes in these settings.

This study is part of the PhD thesis by Elizabeth Tabitha Abbew with funding from the Institute of Tropical Medicine PhD Belgian DGD Scholarship and support from the Johnson and Foundation.

## Supplementary Material


