# Supplementary material for: Prevalence and predictors of NTM in presumed/confirmed drug-resistant TB
Source: IJTLD Open. 2024 Jul 1;1(7):306–13. doi: 10.5588/ijtldopen.24.0242 (PMC11257093; doi:10.5588/ijtldopen.24.0242)
Supplement: Supplementary file 1 [file ijtldopen0242_supplementarydata1.docx]

<http://dx.doi.org/10.5588/ijtldopen.24.0242>

# **Prevalence and predictors of NTM in presumed/confirmed drug-resistant TB**

**Supplementary Table S1:** 16S sequencing, results of MTBc isolates initially missed by MPT =64 assay

| **Case number** | **Spacer** | **Species** |
| --- | --- | --- |
| 2023-02780 | 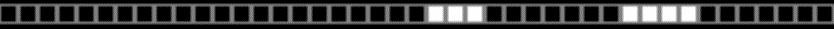 | *M. tuberculosis* Cameroon |
| 2023-02778 | 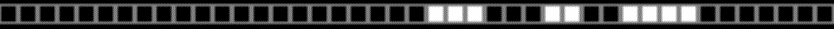 | *M. tuberculosis* Cameroon |
| 2023-02776 | 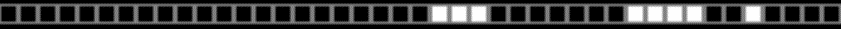 | *M. tuberculosis* Cameroon |
| 2023-02769 | 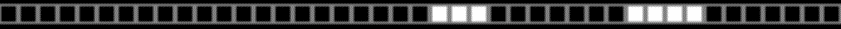 | *M. tuberculosis* Cameroon |
| 2023-02765 | 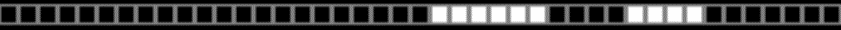 | *M. tuberculosis* Cameroon |
| 2023-02774 | 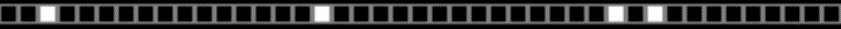 | *M. tuberculosis* Ghana |
| 2023-02772 | 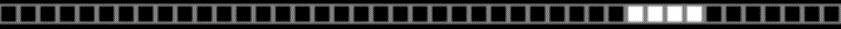 | *M. tuberculosis* Ghana |
| 2023-02770 | 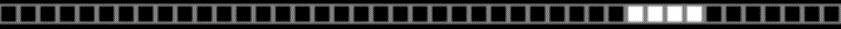 | *M. tuberculosis* Ghana |
| 2023-02767 | 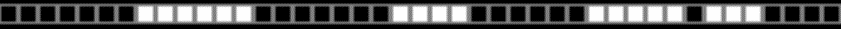 | *M. africanum* West African 1 |
| 2023-02763 | 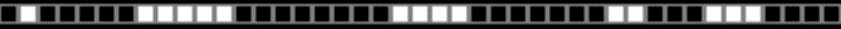 | *M. africanum* West African 1 |
| 2023-02762 | 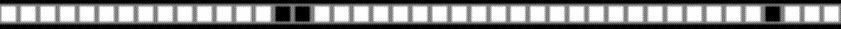 | Incomplete |
| 2023-02764 | 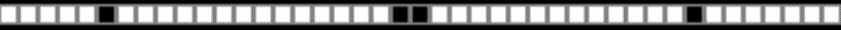 | Incomplete |
| 2023-02766 | 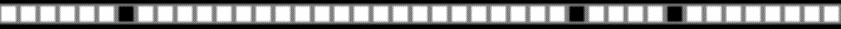 | Incomplete |
| 2023-02777 | 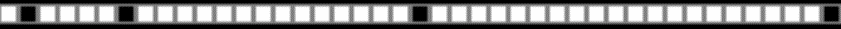 | Incomplete |
